# The Ovicidal, Larvacidal and Adulticidal Properties of 5,5′-Dimethyl-2,2′-Bipyridyl against *Drosophila melanogaster*

**DOI:** 10.1371/journal.pone.0049961

**Published:** 2012-11-20

**Authors:** Matthias B. Van Hiel, Bert Breugelmans, Charles N. Pagel, Adam K. Williams, Aiden K. Varan, Richard Burke, Vernon M. Bowles, Philip Batterham

**Affiliations:** 1 Department of Genetics, Bio21 Molecular Science and Biotechnology Institute, University of Melbourne, Victoria, Australia; 2 Centre for Animal Biotechnology, School of Veterinary Science, University of Melbourne, Victoria, Australia; 3 School of Biological Sciences, Monash University, Clayton, Victoria, Australia; 4 Hatchtech Pty Ltd, Melbourne, Victoria, Australia; Ghent University, Belgium

## Abstract

Insecticide resistance has limited the number of available chemical options for insect pest control. Hence there is a need for new chemistries with novel modes of action. Here we investigate the mode of action for an insecticide that has not yet been released for commercial use. The ovicidal, larvacidal and adulticidal effects of 5,5′-dimethyl -2, 2′-dipyridyl (termed Ha44), which is being developed as a treatment for head lice, were evaluated in the *Drosophila melanogaster* model system. Ha44 demonstrated significant activity against embryos and was capable of arresting development at a number of stages of embryogenesis. The effects of Ha44 on embryos was shown to be reversible following the addition of the metal ions Fe(II) and Fe(III), Cu and Zn. When larvae were exposed to Ha44, lethality was recorded at similar concentrations to those observed for embryos. Using an eYFP reporter system it was shown that Ha44 was able to reduce the levels of both copper and zinc in the digestive tract, confirming the binding of Ha44 to these metals *in vivo*. Ha44 has further been shown to inhibit a zinc containing metalloproteinase *in vitro*. Exposure of adult flies to Ha44 resulted in lethality, but at higher concentrations than those observed for embryos and larvae. The median lethal dose in adult flies was shown to be associated with the type of exposure, with an LD-50 of 1.57 mM being recorded following the direct contact of flies with Ha44, while an LD-50 of 12.29 mM was recorded following the ingestion of the compound. The capacity of Ha44 to act on all stages of the life-cycle and potentially via a range of targets suggests that target site resistance is unlikely to evolve.

## Introduction

Insects represent, by far, the largest number of species in the animal kingdom. Therefore, it is not surprising that in situations where there is competition for resources some insect species have a major detrimental effect on human life. Pest insects can threaten agriculture, human and animal health through direct damage or as vectors for diseases. For several decades insecticides have been the mainstay in attempts to control insect pests, however, the strong natural selection imposed by the widespread use of insecticides has resulted in the evolution of insecticide resistance in at least 500 species [1]. This phenomenon has in-turn caused an increase in insecticide usage with ever decreasing efficacy. Despite exhaustive research, only a limited number of insecticidal chemistries have been developed for commercial release [2]. These insecticides target even fewer proteins [3], [4]. As the vast majority of insecticides target a single insect protein, mutations leading to the appropriate modification or elimination of that protein typically result in very high levels of resistance [5], [6], [7], [8]. New insecticides exploiting novel targets are required to sustain control. To optimize the longevity of such insecticides as control agents, it is desirable that their targets and possible modes of resistance be understood prior to commercial release [9]. Such foreknowledge may be used to stall the evolution of resistance or facilitate improved management if resistance evolves [10]. The capacity to discover the targets and possible modes of resistance for a given insecticide exist. The model insect, *Drosophila melanogaster*, has been used to successfully identify the nature of target-based resistance [9]. Unfortunately, this research has typically been done after the commercial release of the insecticides and, indeed, after resistance to the insecticide has evolved in one or more pest species.

Human head lice, (*Pediculus humanus capitis*) have proven to be a particularly difficult pest to control. This situation is believed to be due to a number of factors including the inability to kill the louse eggs and widespread resistance to many of the commonly used active agents [11], creating the need to identify compounds with novel targets for the control of both the eggs and crawling lice. Metalloproteases required for egg hatching in lice have been suggested as potential new targets [12]. Subsequent research led to the discovery of a series of compounds that prevented egg hatching as well as killing adult lice (unpublished data). Further research identifying the mode of action and the potential for resistance development may be useful in assessing the predicted efficacy of this insecticide in the longer term.

5,5′-dimethyl-2,2′-bipyridyl (termed Ha44) is a heterocyclic organic molecule predicted to chelate heavy metal ions and thereby interact with a range of targets that require heavy metal ions as co-factors within the insect. Ha44 has recently completed a Phase 2 b clinical trial in subjects with head lice, which demonstrated safety and efficacy (unpublished data). This paper analyses the biological activity and mode of action of this compound.

Using both feeding and contact assays in the *D. melanogaster* model system, we demonstrate that Ha44 is lethal to embryos, larvae and adults. We show the ability of Ha44 to inhibit a zinc dependent metalloprotease *in vitro* and provide evidence indicating that Ha44 chelates heavy metals *in vivo*. We show that the lethal effects of the insecticide can be reversed following exposure to the metal ions iron, zinc and copper.

## Materials and Methods

### Drosophila Strains and Food

All flies were raised on standard semolina medium, whereas embryos were reared on apple juice plates (160 ml H_2_O, 1.75 g brewer’s yeast, 5 g agar, 6.5 g sugar, 13 g glucose, 50 ml apple juice, 1.5 ml 10% methylparaben solution). The *w^1118^* line was used as a standard strain of *D. melanogaster* and the *MtnB-eYFP* line, obtained from Dr. Richard Burke was used for the GFP expression analysis.

### Compounds

#### Metal chelators

**Ha44** (5,5′-dimethyl-2,2′-bipyridyl, Sigma), **TPEN** ((N,N,N′N′-tetrakis(-)[2-pyridylmethyl]-ethylenediamine, Sigma) and **EDTA** (Ethylenediaminetetraacetic acid, Sigma). All stock solutions were dissolved in 100% ethanol.

#### Metals

Copper, zinc, iron(II) and iron(III) were supplied by Sigma as CuSO_4_.5H_2_O; ZnCl_2_; FeSO_4_.7H_2_O and FeCl_3_.6H_2_O, respectively and dissolved in distilled H_2_O prior to usage (100 mM).

#### Proteases and inhibitors

**Meprin** 1A (R&D Systems), Bovine **Trypsin** (w/v; Sigma) and a trypsin inhibitor, **AEBSF** 4-(2-Aminoethyl) benzenesulfonyl fluoride hydrochloride (AEBSF; Sigma).

*Substrates:* Azocasein 1% (Sigma) and 10% SDS-PAGE gel containing 0.1% gelatin (Sigma).

### Statistical Analysis

All experiments were set up to include no less than four technical replicates and were repeated at least three times (n ≥4). Results were statistically analysed using GraphPad Prism 5.0 (GraphPad Software, San Diego, CA). For the calculation of the LD-50 and LD-90 values at a 95% significance level and for drafting the dose response curves, the program (PreProbit V1.6.3) was used. The calculation of the dosage-mortality curve was undertaken according to the method described.

### Dose-response Curve Assays for Embryos

Adult flies (*w^1118^* strain) were left to mate in 4 different cages (>200 flies/cage). Freshly laid embryos (0–2 hours) were collected, washed and placed on food plates containing different concentrations of Ha44. In total, 100 embryos per condition (25 embryos from each of the 4 cages) were lined up and incubated at 29°C. The number of hatched embryos was counted after 24 hours of Ha44 exposure and compared to controls. The LD-50 and LD-90 values were calculated as previously described.

### Imaging of the Effects of Ha44 on Embryonic Development

To study the effect of Ha44 throughout embryonic development, the same setup was used as described above. However, for this assay embryos were collected at different time points after laying (2, 4, 6, 8, 10, 14, 16 and 20 hours) and lined up on food plates containing a lethal dose of Ha44 (1 mM) for the remaining time. After 24 hours of incubation at 29°C unhatched embryos were dechorionated (50% bleach; 1 minute). The time of developmental arrest was estimated by imaging under a light microscope.

### Potency Assay: Effect of Metal Chelators on Egg Hatching

Using the same methodology as described for ‘*Dose-response curve assays for embryos’* freshly laid embryos were exposed to different concentrations of Ha44 and two other commercially available metal chelators (TPEN and EDTA). The number of hatched embryos were counted after 24 hours of exposure to a chelator and compared to controls.

### Dose-response Curve Assays for Larvae

For each of seven concentrations of Ha44, 250 first instar larvae were collected and placed on food The LD-50 and LD-90 values were calculated by counting the number of pupae and adults after 10–14 days.

### Dose-response Curve Assays for Adult Flies

- Contact assay
  Sexually mature non-virgin females (4 days post-eclosion) were exposed in a contact assay over a period of 24 hours to different doses of Ha44. Glass scintillation vials and plugs were coated with 300 µl of acetone containing different concentrations of the compound. After evaporation of the liquid by rolling, the vials were plugged with a coated piece of cotton wool. The cotton wool plugs were wrapped into parafilm® after being soaked in 5% sucrose and prior to exposure to acetone. After evaporation of the acetone, the parafilm® was punctured multiple times with a needle to allow the flies to access the sucrose liquid. Subsequently, 20 flies were placed in each vial. 24 hours later, mortality was scored and compared to controls. The LD-50 and LD-90 values were calculated and expressed in mM/vial.
- Ingestion assay
  Sexually mature females (4 days post-eclosion) were fed different concentrations of Ha44 over a period of 48 hours. Flies were transferred into small vials (2 cm in height with a diameter of 4 mm) of which the lid held 500 µl of food containing different concentrations of Ha44 (10 flies/cage). Mortality was scored after 48 hours and the LD-50 and LD-90 values were calculated.

### *In vitro* Activity Assays

- Activation of Meprin
  Recombinant mouse meprin 1A was activated according to the manufacturer’s instructions. Briefly, inactive meprin in activation buffer (Tris pH 7.5 [50 mM], NaCl [150 mM] 0.05% Brij-35) was incubated in the presence of 0.01% trypsin for 2 hours at 37°C, after which trypsin activity was inhibited by the addition of AEBSF to a final concentration of 1 mM.
- Gelatin zymography
  Samples of active meprin 1A (100 ng) in non-reducing SDS-PAGE buffer were resolved through 10% SDS-PAGE gel containing 0.1% gelatin (Sigma Aldrich). After electrophoresis, marker lanes were cut off and fixed in 50% methanol (v/v) and 10% (v/v) acetic acid in deionised water. The remaining gel was washed twice in Triton-X (2.5%; Sigma Aldrich) in deionised water. Individual lanes were cut from the gel and were incubated overnight at 37°C in a buffer consisting of Tris pH 8.0 (0.1 M), NaCl (0.2 M) and Triton-X (0.02%; v/v) in the presence or absence of Ha44. Gel strips were then fixed, as described above, and stained with Coomassie Brilliant Blue R-250 (0.1% [w/v]; Sigma Aldrich) for 2 hours, prior to destaining in 10% methanol (v/v) and 10% (v/v) acetic acid in deionised water.
- Azocasein assay
  Activity of meprin 1A in the presence of Ha44, TPEN and EDTA was measured using an azocasein assay as previously described [13]. Briefly, azocasein (1% [w/v]) in meprin activation buffer, was pre-incubated with inhibitor at different concentrations for 10 minutes at 37°C. Activated meprin 1A (100 ng) was then added to 100 µl of the pre-incubated azocasein solution containing Ha44. Samples were then incubated at 37°C for 2 hours. Uncleaved azocasein was precipitated by the addition of 75 µl of trichloroacetic acid (10% [w/v]) prior to incubation on ice for 10 minutes. Samples were centrifuged at 13000 rpm for 10 minutes and 90 µl of each of the supernatants was removed, combined with 14 µl NaOH (5 M) and the absorbance at 405 nm was read using a Labsystems Multiskan MS plate reader (Thermo Electron Corp). Data was blank subtracted and plotted. A sigmoid dose response curve was fitted using the non-liner regression function of GraphPad Prism 5.0.

### Metal-compound Interaction Assays

Freshly laid embryos were exposed to food containing a lethal dose of Ha44 and a 1 mM concentration of one of the four metals [Cu, Zn, Fe(II) and Fe(III)] or a mix of all four metals (0.25 mM/metal). After 24 hours, the number of hatched embryos was counted and compared to three controls: (*i*) no exposure to Ha44 or metals, (*ii*) exposure to Ha44 but no exposure to metals and (*iii*) exposure to metals.

A similar method was used to study the effect of metals on larvae exposed to a lethal dose of Ha44. First instar larvae were placed on plates containing Ha44 and a single metal or a mix of the four metals. Subsequently, the number of pupae and successfully eclosed adults were counted and compared to controls in which larvae were not exposed to the compound and/or metal(s).

### GFP Expression Analysis and Microscopy

The *MtnB-eYFP* line contains a metallothionein promoter region (MtnB) linked to a reporter region (eYFP). For the eYFP reporter analysis, the *MtnB-eYFP* line was crossed to a *w^1118^* line and the flies were allowed to deposit eggs on food plates. The offspring were raised to third instar larvae under different experimental conditions. Larvae were reared on: (*i*) one of the four metals (1 mM), (*ii*) Ha44 (0.1 mM), (*iii*) TPEN (0.1 mM), (*iv*) one of the four metals in combination with Ha44 and (*v*) one of the four metals/the mix in combination with TPEN. Next, the midgut was dissected and analysed under a fluorescence microscope (Olympus SFX12) at a 25 times magnification and an equal exposure time.

### Effects of Ha44 on Fertility and Mating Behaviour

By means of a contact assay, freshly eclosed virgin male and female flies were exposed to a sub-lethal dose of Ha44 (0.5 mM) for 72 hours. Subsequently, virgins were mated to unexposed sexually mature flies and the eggs and progeny were counted and compared to controls.

The effect of Ha44 on mating behaviour of male flies was studied by means of a contact assay. Sexually mature males were exposed to a lethal dose of Ha44 (4 mM) for 3 hours. Next, the exposed flies were placed with virgin females (1∶1 ratio) in small vials containing food. Courting and mating behaviour of the effected males (wing movement, circling the female and mounting) were scored during the first hour and the couples were left to mate for three days onwards. The embryos were collected every 12 hours. Hatching numbers were counted and compared to controls.

## Results

### Effects of Ha44 on Embryonic Development

#### Dose response effect of Ha44 on hatching

**Figure 1:**
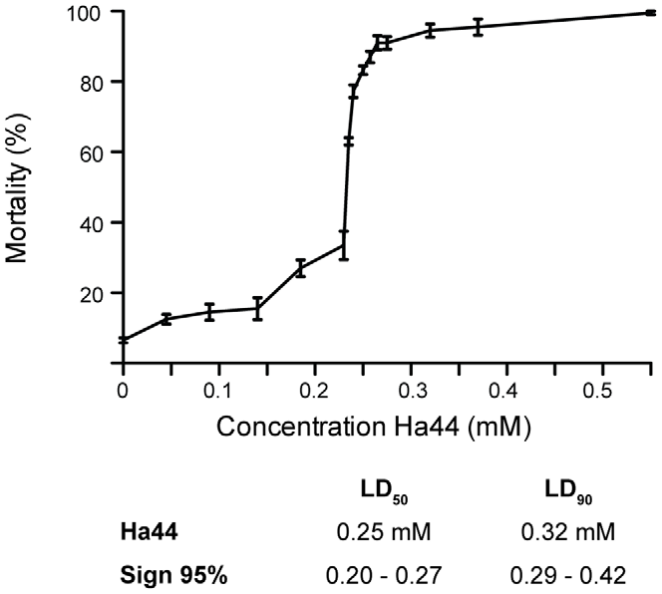
Dose response effect of Ha44 on hatching. Concentration of Ha44 (mM) to which 0–2 hours old embryos were exposed for 24 hours at 29°C (100 eggs/concentration). For each concentration the average mortality (percentage) is presented±S.E. The legend below the graph displays the LD-50 and −90 values (mM) for Ha44 at a 95% significance level according to Probit.

Exposing 0–2 hour embryos to a concentration of Ha44 (0–0.1 mM) over a period of 24 hours had no significant effect on hatching (Fig. 1). However, increasing the dosage to 0.3 and 0.6 mM resulted in a significant negative effect on hatching with a mortality of up to 100% (Fig. 1). Using Probit, the LD-50 and LD-90 values were calculated at concentrations of 0.25 mM and 0.32 mM, respectively, indicating an increase in mortality by 40% within a narrow range -0.07 mM (Fig. 1).

#### Effect of the Ha44-exposure time on hatching

**Figure 2:**
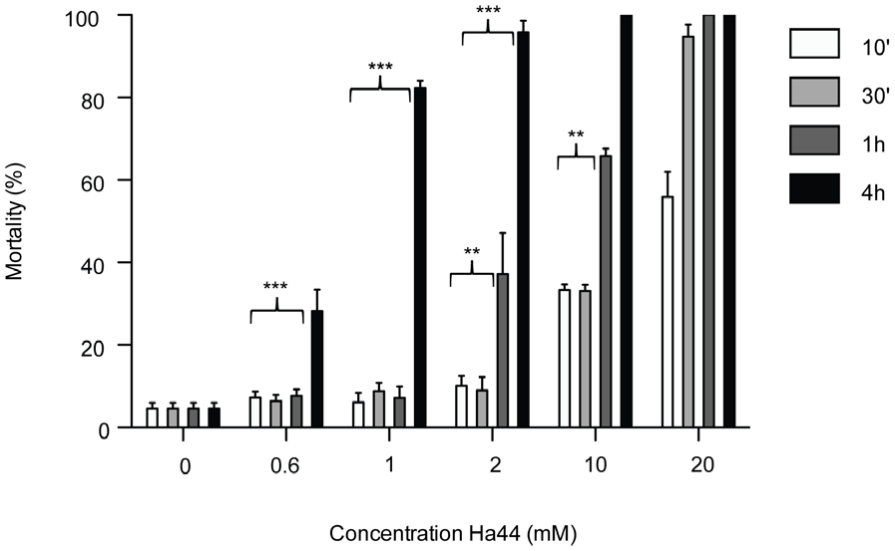
Effect of Ha44 exposure time on hatching. Concentration of Ha44 (mM) to which 0–2 hour old embryos were exposed over different periods of 10 minutes to 4 hours at 29°C (100 eggs/condition). For each concentration/exposure time the average mortality (percentage) is presented (n = 4)±S.E. and corresponds to the number of non-hatched embryos after 24 hours. Possible significant differences between the four exposure times were assessed using a two-way ANOVA model combined with a Bonferroni post hoc test (** = p<0.001; *** = p<0.0001).

The effect of exposure time in relation to the Ha44 concentration was determined by exposing embryos to different concentrations of Ha44 over varying time points: from 10 minutes up to four hours (Fig. 2). Increasing the exposure time had a significant negative effect on hatching and was positively correlated to the Ha44 concentration (p<0.0001). A two-way ANOVA (using a Bonferroni correction for multiple comparisons) demonstrated significant differences between embryos that were exposed for four hours to 0.6 and 1 mM of the compound, and the shorter exposure times (10′ to 1 hour) at the same dosage (p<0.0001). An increase in the Ha44 dosage to 2 and 10 mM resulted in a significant negative effect on hatching after one hour (p<0.001). At the highest concentration (20 mM) over 60% of the embryos did not hatch after a 10-minute exposure time, whereas a longer exposure resulted in approximately 100% mortality compared to controls (p<0.0001).

#### Visualizing the effects of Ha44 during embryonic development

Embryos were exposed to a lethal dose of Ha44 (1 mM) at different time points during development over a period of 24 hours. The non-hatched embryos were then dechorionated and the time of developmental arrest was visualized. Ha44 was able to arrest development in early stages of embryogenesis (Stage 5: blastoderm stage), at 12–15 hours (Stages 8–10: germ band elongation) and at a very late stage (Stages 16–17) near the point of hatching (data not shown).

#### Metals inhibit the effect of Ha44 on hatching

**Figure 3:**
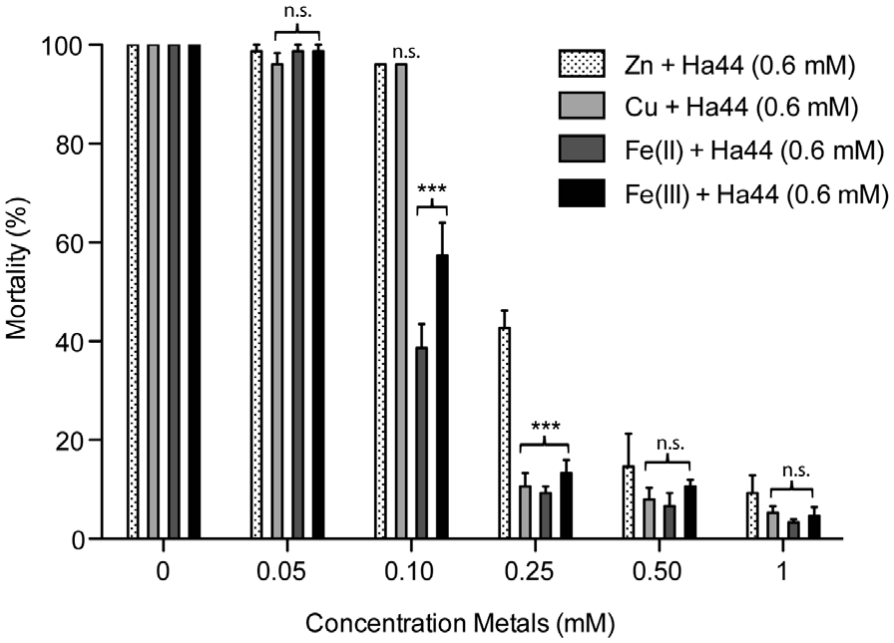
Effect of different metals on hatching of Ha44-exposed embryos. Freshly laid embryos (0–2 hours) were exposed to a lethal dose of Ha44 in the absence/presence of metals [Cu, Zn, Fe(II) and Fe(III)] at different concentrations (mM). Possible significant differences in potency between the different metals were assessed using a two-way ANOVA model combined with a Bonferroni post hoc test (*** = p<0.0001, n.s. = not significant).

Next, we studied the ability of different metals [Cu, Zn, Fe(II) and Fe(III)] to reverse the lethal effect of Ha44 on developing embryos. As shown in Figure 3, a low concentration (0.05 mM) of metal(s) did not reverse the lethal effect of Ha44 (0.6 mM) on developing embryos. A two-way ANOVA (using a Bonferroni correction for multiple comparisons) demonstrated that increasing the metal concentration to 0.1 mM, iron [Fe(II) and Fe(III)] significantly decreased the effect of Ha44 on hatching compared to controls. Adding the same concentration of zinc or copper to embryos that were exposed to Ha44 had no significant effect on hatching compared to controls. At a dose of 0.25 mM, Fe(II), Fe(III) and copper decreased the lethal effect of Ha44 on embryos by over 80%, while the effect of zinc was significantly lower (p<0.0001). At a relatively high dose (1 mM) all four metals reduced the effect of Ha44 on developing embryos to 90–95% of the value of the controls.

### Comparing the *in vitro* and *in vivo* Effect of Metal Chelators

**Figure 4:**
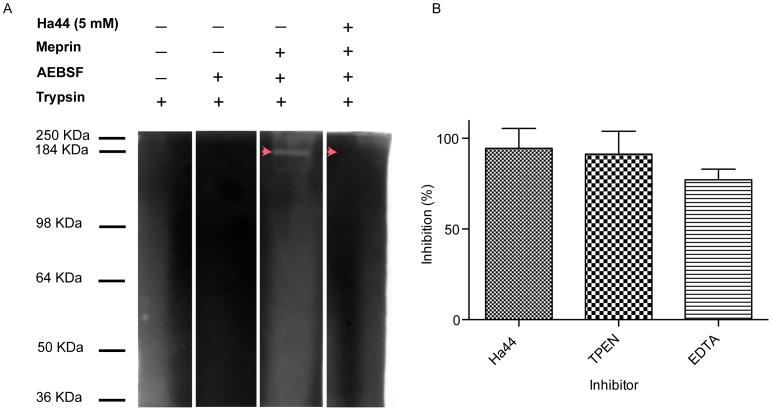
*In vitro* inhibitory potency of Ha44: **Panel (A).** zymogram. Lane 1 and 2 are controls, in lane 3 active meprin 1A is visualised (148 KDa) and lane 4 displays the inhibitory effect of Ha44 on meprin 1A. **Panel (B):** azocasein (1%) assay. Inhibitory effect (%) of Ha44, TPEN and EDTA (1 mM) on activated meprin 1A compared to a control condition (0% inhibition).

- In vitro potency of Ha44 compared to other metal chelators
  Zymography was used to determine the capacity of Ha44 to inhibit the Zn dependent metallo-protease (meprin-1A) which has a similar protease domain to previously described hatching enzymes (Fig. 4A). In addition the *in vitro* inhibitory potency of the compound was determined by means of a chromogenic substrate assay (azocasein) and compared to the commercial metal chelators: TPEN [binds Fe, Cu, Zn but not Ca [14] and EDTA [most commonly used metal chelator [15].
  As illustrated in Figure 4A, the presence of a 148 kDa band in lane 3 and the absence of this band in lane 4 on the zymogram, show that Ha44 was able to completely inhibit the proteolytic activity of meprin-1A *in vitro* at the concentration of 5 mM. In addition, Figure 4B illustrates that Ha44 was as potent an inhibitor as TPEN and EDTA.
- In vivo potency of Ha44 and other metal chelators on hatching
  Freshly laid embryos were exposed to different concentrations of Ha44 and two other commercially available metal chelators (TPEN and EDTA). Only Ha44 could prevent hatching in embryos at a relatively low dose (0.25 mM; Fig. 1). TPEN and EDTA on the contrary, could not prevent hatching, not even at ten and hundred fold higher doses (2.5 and 25 mM) (data not shown).

### Effects of Ha44 on Developing Larvae

#### Dose response effect of Ha44 on developing larvae

**Figure 5:**
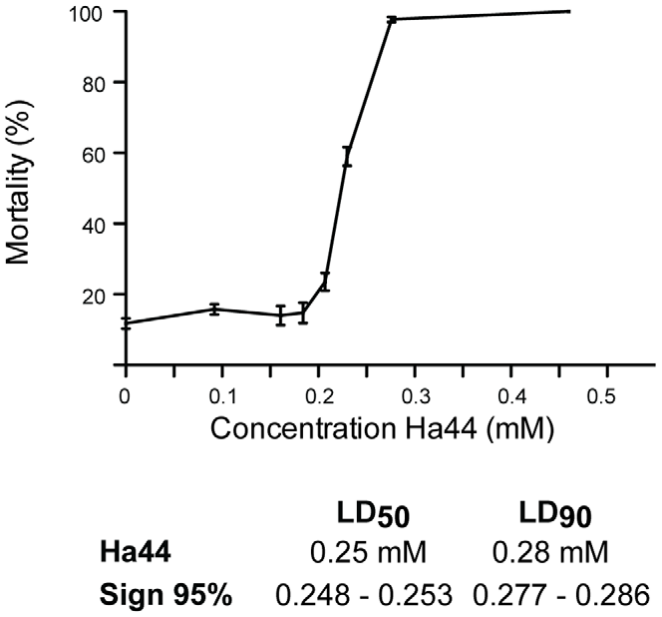
Dose response effect of Ha44 on larval development. Concentration of Ha44 (mM) to which first instar larvae were exposed in function of mortality (50 larvae/condition). For each concentration the average mortality (percentage) is presented±S.E. The legend below the graph displays the LD-50 and −90 values (mM) for Ha44 at a 95% significance level according to Probit.

First instar larvae were exposed to a range of concentrations of Ha44 and the effects on development, pupation and eclosion determined. As illustrated in Figure 5, doses lower than 0.2 mM had no significant effect on the development of larvae. Using Probit, the LD-50 and LD-90 values were calculated at a concentration of 0.25 mM and 0.28 mM, respectively, indicating an increase in mortality by 40% within a narrow range - 0.03 mM (Fig. 5).

#### Metals inhibit the effect of Ha44 on developing larvae

**Figure 6:**
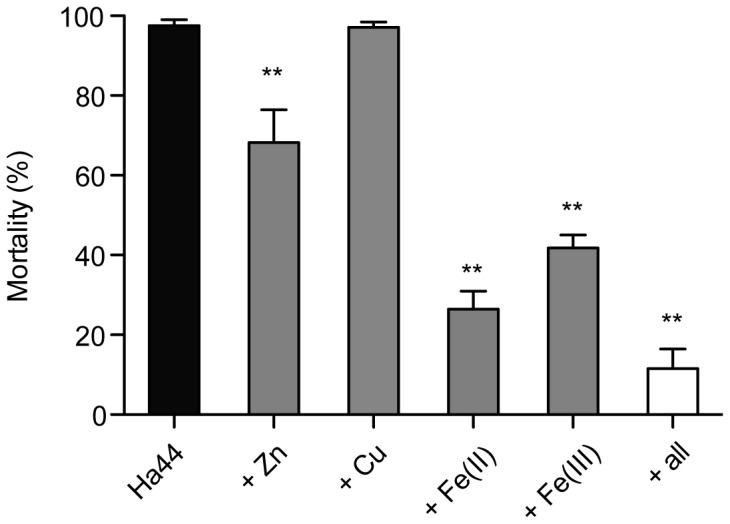
Effect of different metals on Ha44-treated developing larvae. First instar larvae laid were exposed to a lethal dose of Ha44 in the absence/presence of metals (1 mM) [Cu, Zn, Fe(II), Fe(III) and a combination]. A non-parametrical, Mann Whitney test (One-tailed) was used to prove significant differences in the inhibitory potency between the metals (grey and white bars) and the control condition (Ha44, black bar) (** p<0.01)±S.E.

We studied the ability of different metals [Cu, Zn, Fe(II) and Fe(III)] to inhibit the insecticidal effect of Ha44 on developing larvae. As shown in Figure 6, copper was not able to reverse the lethal effect of Ha44 on developing larvae. On the contrary, iron [Fe(II)/Fe(III)] and zinc as well as the combination of all four metals, could significantly decrease the negative effect of Ha44 on larvae compared to the controls (p<0.01). At lower concentrations Fe(II) rescued up to 70% of the Ha44-treated larvae, while Fe(III) and zinc required greater concentrations to rescue up to 50% and 25% of the larvae, respectively. The combination of all four metals reduced approximately 90% of the negative effect of Ha44 on developing larvae.

#### Ha44 inhibits Zn and Cu metals in the gut

**Figure 7:**
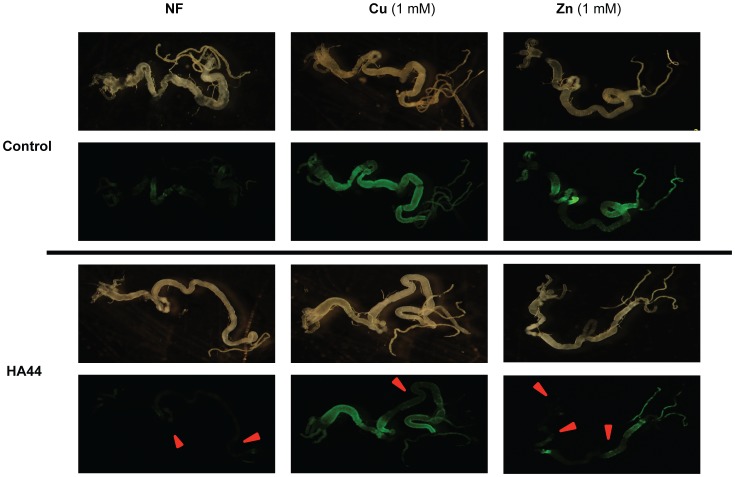
An *MtnB-eYFP* reporter (green fluorescent signal) indicates relative copper and zinc content in midguts from dissected third instar larvae. Larvae were reared on normal food (NF) or food containing 1 mM Cu or Zn. Expression of eYFP was inhibited by addition of 0.1 mM Ha44. Red arrows indicate regions with lower eYFP expression. Pictures of every condition were taken with light (top) and fluorescence filter (bottom).

The metallothionein promoter (MtnB) is activated by high intracellular concentrations of heavy metals. Here MtnB was used to drive the expression of the enhanced yellow fluorescence reporter protein (eYFP). Copper or zinc induced strong expression throughout the midgut of third instar larvae (Fig. 7). Iron did not induce the MtnB promoter (data not shown). In all cases a dose of Ha44 (0.1 mM) was able to decrease the signal of eYFP (Fig. 7, lower 6 panels) indicating that Ha44 effectively made metals less available *in vivo*. TPEN was used as a positive control and gave similar results to Ha44 (data not shown).

### Effects of Ha44 on Adult Flies

#### Dose response curves of adult flies exposed to Ha44 in food and through contact

**Figure 8:**
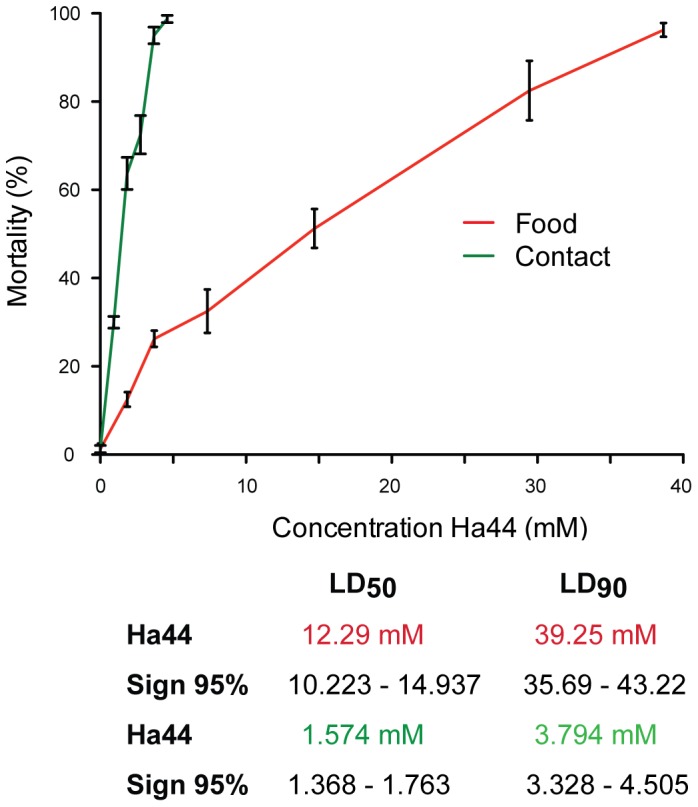
Dose response effect of Ha44 on adult flies (contact versus ingestion). Concentration of Ha44 (mM) to which adult flies were exposed in function of survival after 24 hours. The average mortality (percentage) is presented±S.E. for each concentration and both experimental conditions; contact (green line) and ingestion (red line). The legend below the graph displays the LD-50 and −90 values in mM for Ha44 for both conditions (red and green) at a 95% significance level according to Probit.

Sexually mature adult flies were exposed to a range of concentrations of Ha44 over a period of 24 hours and the numbers of survivors were counted. We compared the dose response when Ha44 was administered via either contact or ingestion. Using a two-way ANOVA (using a Bonferroni correction for multiple comparisons) we demonstrated significant differences in LD-50 values for these two methods of exposure (Fig. 8). Dose response curves and regression lines were calculated and the LD-50/90 values were predicted. For flies that had been in contact with Ha44 the LD-50 value was estimated at a dose of 1.57 mM (Fig. 8, green numbers). Furthermore, flies exposed to Ha44 at [4 mM] through contact for one hour displayed behavioural changes including disorientation, an inability to fly or to climb the walls of the vials and lay on their backs. The LD-50 for flies that were exposed to Ha44 through ingestion was approximately eight fold higher (12.29 mM) than for flies that had been in contact with the compound (Fig. 8, red numbers).

#### Effects of Ha44 on fertility and mating behavior

To study the short-term effect of Ha44 on mating behaviour, sexually mature males were exposed to Ha44 [4 mM] for 3 hours after which they were each placed with a single female. Contrary to the controls, the exposed males displayed no courting or mating behaviour (wing movement, circling the female) in the following 24 hours. However, 48 hours after exposure males did start to display these behaviours and viable offspring were produced from the matings (data not shown).

The long-term effect of Ha44 on fertility was studied on adult flies by means of contact assays. Freshly eclosed virgin male and female flies that had been exposed sub-lethal dose of Ha44 (0.5 mM) for 72 hours and mated to unexposed sexually mature flies did not show a significant decrease in fertility compared to a controls (data not shown).

## Discussion

### Ha44 has a Lethal Effect on all Life Stages

The need to identify new safe and effective insecticides with novel modes of action represents a constant challenge as insect pests have demonstrated a remarkable ability to become resistant to many of the currently available insecticides. This situation is equally true for head lice where resistance has been reported to a number of the commonly used actives. At the present time, the compound 5,5′-dimethyl-2,2′-bipyridyl is being investigated as a potential new therapeutic treatment for head lice control, recently completing a Phase 2 b clinical trial (Clinical trials.gov identifier: NCT01336647). Further research identifying the mode of action and the potential for resistance development with this compound may be useful in assessing the efficacy of this insecticide in the longer term. This study describes the insecticidal activity of the metal chelating compound 5,5′-dimethyl-2,2′-bipyridyl (Ha44, MWt 184) against all life stages of *D. melanogaster*. The most notable feature of the compound was Ha44’s ability to prevent egg hatching. This result implies that Ha44 can effectively penetrate the eggshell, which is impervious to most compounds, to quickly kill developing embryos. Even small molecules such as sodium hypochlorite (MWt 74.5), routinely used for removal of the chorion, are unable to pass through the thin waxy layer just underneath the endochorion [16]. We have demonstrated that Ha44 is 100% lethal to embryos in a concentration and time dependent manner. The intact nature of the chorion suggests that Ha44 passes unhindered through the eggshell into the embryo where it disrupts development. In addition, lethality was observed at all stages of embryonic development. This is a valuable insecticidal property not observed for the two other known metal chelators (TPEN and EDTA) tested in this study. EDTA has previously been shown to be non-ovicidal against eggs of the sheep blowfly (*L. cuprina*) and the human body lice (*Pediculus humanus corporis*) [12], [17]. The inability of EDTA and TPEN to disturb embryonic development may be due to a number of factors including a failure to penetrate the egg or to a difference in the rate that these metal chelators can either inhibit specific metal dependent proteins or bind to metals that are important for embryo development and hatching. Both *in vitro* and *in vivo* testing has shown that Ha44 is a potent ovicide against human head lice (*Pediculus humanus capitis* unpublished results). The ovicidal action of a compound is an extremely important phenomenon when assessing its potential value for controlling head lice. Indeed the general lack of ovicidal compounds has been the key reason for the need for current generation insecticides to be applied twice (7–10 days apart) to kill newly emerged nymphs [18].

Ha44 was also shown to be lethal to larvae at a similar dose to that observed for embryos. However, it is worth noting that since the development of *Drosophila* larvae takes approximately 10 days compared to 20–24 hours for embryos, larvae were exposed ten times longer to the compound. In addition, larvae were not only exposed to Ha44 through contact as for embryos, but also through feeding. The observation of distinct discolouration of the salivary glands and the cuticle of third instar larvae treated with Ha44 suggested that Ha44 may be taken up in larvae via the digestive tract as well as via the cuticle (unpublished results).

By comparing the difference in the effects of the compound between contact and ingestion in adult flies, we observed that an eight-fold increase in concentration of compound was required to induce a lethal effect when the compound was mixed in food compared to when flies were exposed to the compound through contact only. The observed difference in potency indicates that Ha44 is more lethal when taken up via the cuticle than when taken up via the digestive tract. Possible reasons for this apparent difference in potency include metabolism of Ha44 or inactivation of the compound via binding of free bivalent metals present in the food. It was of interest that, males who were briefly exposed to the compound did not engage in courting behaviour or sexual intercourse until they recovered from the effects of exposure, approximately 48 hours later. Longer term exposure of adult flies to a sub-lethal concentration of the compound did not decrease fertility in males and females, both sexes recovered, after which they mated and produced viable and fertile offspring.

### Mechanism of Action of 5,5′-dimethyl-2,2′-bipyridyl

In order to more fully understand the nature of the interaction between Ha44 and different metal ions, experiments were conducted on both embryos and larvae to determine if the lethal effects of this compound could be reversed by the addition of specific metal ions. Data obtained indicated that indeed it was possible to reverse the lethal effect of Ha44 on *Drosophila* embryos by adding a sufficient amount of zinc, copper or iron to the food mixture. In the case of copper, it was interesting to note that while the addition of copper was able to rescue embryos, it was not able to rescue larvae from the effects of Ha44. This implies that Ha44 is able to inhibit important Cu containing enzymes/pathways within the larvae that are either absent or redundant in the developing embryo, thereby enabling rescue. Furthermore, we showed that Ha44 binds metals in the digestive tract of adult flies using an eYFP reporter signaling system; the fluorescent signal that is induced by the presence of metals such as copper and zinc in the digestive tract is reduced significantly when Ha44 was added to the food of adult flies. Therefore, the lethal effects of Ha44 that where observed on the various stages of the insect are likely due to the ability of Ha44 to interfere with metal dependent proteins in the insect.

Previous research had indicated a potential role for metalloproteases in the hatching of lice [12]. Given the requirements of these enzymes for metal ions, we investigated whether Ha44 could inhibit a metal-dependent enzyme. The inhibition by Ha44 of the zinc dependent metalloendopeptidase was of interest since the Meprins have a similar protease domain structure to members of the Astacin family of proteases that are known to be involved in egg hatching in a number of species [19] and hence are a potential candidate for Ha44 inhibition. Meprin-1A, a zinc-dependent mouse metalloendopeptidase, was inhibited by relatively high concentrations of Ha44. Whether the inhibition of meprin-1A by Ha44 is due to the actual removal of the metal ions or to binding to the active site (or a combination of both) is yet unclear. Some chelating agents are known to be capable of stripping metals from their binding sites in enzymes [20], [21], [22], so it is possible that this is one means by which Ha44 is exerting its effects in *Drosophila*. Given the chelating properties of Ha44 and the importance of the metal ions (Fe, Zn and Cu) in biological systems one might predict this chemical to be insecticidal throughout development. The fact that we did not observe these effects with the other metal chelators tested could be due to several reasons including an enhanced ability of Ha44 to penetrate the insect to reach its respective targets or the efficiency with which Ha44 chelates metals associated with protein targets.

### Potential Implications for Resistance to 5,5′-dimethyl-2,2′-bipyridyl

Of major concern in insect pest management is the occurrence of resistance imposed by the widespread use of an insecticide. In the case of head lice resistance to permethrin, pyrethrin and malathion has been described [11], [23], [24]. More recently spinosad and ivermectin have received approval in the USA for use in controlling head lice. Resistance to these insecticides has evolved in other insect species [25], [26], [27], [28]. High level resistance to spinosyn has been associated with loss of function mutations in a gene that encodes the target, the nicotinic acetylcholine a6 receptor (nAChR-alpha-6) subunit [9]. While loss of function mutations are likely to arise frequently in pest populations, it remains to be determined if nAChR-alpha-6 is the target of spinosyn in head lice.

The potential for Ha44 target site resistance to evolve is predicted to be low. Ha44 is a chelator of at least three different bivalent metals (Fe, Cu, Zn) and the activity of a vast number of proteins (many of which are required for viability) is dependent on these metals. Therefore, it is extremely unlikely that the lethality of Ha44 is caused by the loss of function of a single metalloprotein. Ha44 probably has a range of targets and it is unlikely that a mutation in a gene encoding any one of these targets could confer a significant level of resistance. A mutation in any one gene could, at best, only make one target insensitive to Ha44; all other potential target proteins would remain sensitive.

For most insecticides the highest levels of resistance are associated with target site insensitivity, which has been a major driver for the constant need to develop new insecticides to replace those that have been rendered ineffective via this mechanism [10]. For Ha44 two other mechanisms that have the potential to confer lower levels of resistance need to be investigated. Based on the impact of added Cu, Zn and/or Fe to Ha44 efficacy, mutations that result in an increased uptake or retention of these metals may confer resistance, as could increased metabolism of Ha44. The first possibility could be tested by modulating the levels of expression of genes involved in heavy metal homeostasis using transgenic strains of *D. melanogaster*. Given the broader physiological implications, the fitness costs of such modulations should be assessed. The second possibility could be tested in strains *D. melanogaster* overexpressing metabolic genes when exposed to Ha44.

With existing options for the control of insect pests such as human head lice being diminished by resistance to current generation insecticides, new chemicals with novel targets need to be developed. To avoid the constant need to replace insecticides due to resistance there is a need to understand the mode of action of insecticides before they are deployed for insect pest control [9]. In this study, the biological activity and mode of action of Ha44 has been investigated. This agent is effective throughout the lifecycle of this model insect potentially targeting Cu, Zn and Fe dependent proteins, and using this information, the predicted resistance can be assessed.
